# Respuesta a la carta al editor «Poniéndole la lupa a la estrategia farmacoinvasiva»

**DOI:** 10.47487/apcyccv.v5i2.368.

**Published:** 2024-06-24

**Authors:** L. Marco Lopez-Rojas, Cynthia Paola Paredes Paucar, Paol Rojas De La Cuba, W. Germán Yábar Galindo, Jorge Orlando Martos Salcedo, Piero Custodio-Sánchez

**Affiliations:** 1 Hospital Nacional Alberto Sabogal Sologuren - EsSalud. Lima, Perú. Hospital Nacional Alberto Sabogal Sologuren - EsSalud Lima Perú; 2 Hospital Germans Trias i Pujol, Unidad de Insuficiencia Cardiaca. Barcelona, España. Hospital Germans Trias i Pujol Unidad de Insuficiencia Cardiaca Barcelona España; 3 Hospital Nacional Guillermo Almenara Irigoyen-EsSalud. Lima, Perú. Hospital Nacional Guillermo Almenara Irigoyen-EsSalud Lima Perú; 4 Hospital Regional Docente de Cajamarca-MINSA. Servicio de Cardiología. Cajamarca, Perú. Hospital Regional Docente de Cajamarca-MINSA Servicio de Cardiología Cajamarca Perú; 5 Hospital Nacional Almanzor Aguinaga Asenjo - EsSalud. Unidad de cardiología intervencionista. Chiclayo, Perú. Hospital Nacional Almanzor Aguinaga Asenjo - EsSalud Unidad de cardiología intervencionista Chiclayo Perú

*Sr. Editor*

Agradecemos al Dr. Frank Britto por el comentario a nuestro artículo: «Propuesta de manejo inicial del infarto de miocardio con elevación del segmento ST no complicado en centros sin capacidad de intervención coronaria percutánea en el Perú» publicado en el último número del 2023 de la revista ^1^.

Al respecto, compartimos que la evidencia ha mostrado que la estrategia farmacoinvasiva, realizada en tiempos adecuados, es el abordaje más favorable en centros sin capacidad de ofrecer una intervención coronaria percutánea (ICP) primaria oportuna ^2^, como son la mayoría de los centros públicos en nuestro país. En esta estrategia, la ICP temprana de rutina entre las 2 - 24 h tras una fibrinolisis exitosa es segura y efectiva (IA), al estar asociada con reducción de isquemia recurrente, reinfarto y falla cardiaca frente a la fibrinolisis aislada y, por otro lado, la ICP de rescate, realizada de emergencia tras una fibrinolisis fallida (IA), está asociada con una reducción de eventos mayores (muerte, reinfarto, evento cerebrovascular o falla cardiaca severa) frente a solo un manejo conservador ^3^^,^^4^.

El metanálisis de Fazel indica mayor probabilidad de muerte en aquellos pacientes que reciben solo fibrinolisis frente a una estrategia farmacoinvasiva ^2^; además, es de resaltar que un registro nacional no encontró diferencias significativas en los resultados intrahospitalarios entre aquellos sometidos a estrategia farmacoinvasiva frente a aquellos que recibieron ICP primaria ^5^, un estudio latinoamericano mostró que la estrategia farmacoinvasiva es más costo-efectiva en relación a la ICP primaria y la no reperfusión ^6^, y tanto la guía europea como el posicionamiento latinoamericano más reciente recomiendan la transferencia inmediata de todos los pacientes después de la fibrinolisis hacia centros con capacidad de ICP ^3^^,^^7^.

Sin embargo, ante la interrogante planteada, de un escenario que sucede con frecuencia en nuestro deficiente sistema de salud, ¿qué conducta tomar si por cualquier problema el centro que realiza la fibrinolisis exitosa no puede transferir al paciente dentro de las primeras 24 h para la ICP? En este contexto, la evidencia científica disponible, proveniente principalmente de estudios observacionales (Tabla 1), indica resultados similares de la ICP luego de las 24 h respecto a la ICP en las primeras 24 h tras una fibrinolisis exitosa, y respaldan buscar transferir siempre a todos los pacientes, para completar la estrategia farmacoinvasiva, lo más pronto posible, independientemente de sus características individuales ^8^^-^^15^ ya que tienen riesgo de isquemia recurrente y reinfarto que podrían conducir a menor función ventricular y mayor probabilidad de muerte ^16^^,^^17^.

**Tabla 1:** Estudios de pacientes sometidos a intervención coronaria percutánea (ICP) posterior a las veinticuatro horas tras una fibrinolisis exitosa

Fuente (año de publicación)	Métodos	Resultados
Feasibility and efficacy of delayed pharmacoinvasive therapy for ST elevation myocardial infarction. (2023) ^8^	Registro prospectivo. Pacientes fibrinolizados y arterias no completamente ocluidas en angiografía. Grupo 1: ICP 3 - <= 24 h tras fibrinolisis Grupo 2: ICP 24 - 72 h tras fibrinolisis	183 en grupo 1 y 154 en grupo 2. Los eventos adversos cardiovasculares mayores (muerte, rehospitalización por reinfarto, falla cardiaca congestiva, revascularización y stroke) fueron de 8,7% en el grupo 1 y 12,9% en el grupo 2 ( p =0,152), y no se encontraron diferencias significativas en los resultados individuales.
Post-streptokinase PCI in STEMI patients exceeding the 24-h guidelines. (2021) ^9^	Estudio observacional. Pacientes fibrinolizados con éxito con estreptoquinasa. Grupo 1: ICP <= 24 h. Grupo 2: ICP > 24 h - 1 semana.	129 pacientes, 57 en grupo 1 y 72 en grupo 2. No fueron detectados puntos primarios (compuesto de muerte, falla cardiaca congestiva y reinfarto) en ambos grupos hasta los 30 días. No se encontraron diferencias estadísticas significativas entre los grupos respecto a FEVI, dimensiones del ventrículo izquierdo, complicaciones y flujo TIMI.
GRACE score and cardiovascular outcomes prediction among the delayed coronary intervention after post-fibrinolytic STEMI patients in a limited PCI-capable Hospital. (2020) ^10^	Cohorte retrospectiva. Pacientes fibrinolizados con éxito, que fueron sometidos a una intervención coronaria retrasada (24 h a 2 semanas).	229 (67.2%) con GRACE bajo (< 126 puntos), y 112 (32,8%) con GRACE intermedio - alto (>= 126 puntos). El compuesto primario (muerte, rehospitalización por síndrome coronario agudo, rehospitalización por falla cardiaca y stroke) a 30 días fue de 2,2% en grupo de GRACE bajo y 11,6% en el grupo de GRACE intermedio - alto (p=0,001), y a 6 meses fue de 3,9% y 13,4% respectivamente (p= 0,003)
Cardiovascular outcomes of early versus delayed coronary intervention in low to intermediate-risk patients with STEMI in Thailand: a randomised trial. (2019) *^11^	Estudio aleatorizado. Pacientes fibrinolizados con éxito, con GRACE bajo a intermedio (< 155). Grupo 1: ICP 3 - 24 h. Grupo 2: ICP > 24 h.	81 pacientes en cada uno de los grupos. A 30 días, el resultado cardiovascular compuesto (muerte, rehospitalización por síndrome coronario agudo, rehospitalización por falla cardiaca y stroke) fueron 4,9% en el grupo 1, y 2,5% en el grupo 2 (p=0,682). A 6 meses, el compuesto cardiovascular fue de 16,1% en el grupo 1 y 6,2% en el grupo 2 (p=0,054).
Timing of Coronary Angiography After Successful Fibrinolytic Therapy in ST-Segment Elevated Myocardial Infarction. (2019) ^12^	Estudio prospectivo. Pacientes fibrinolizados con éxito, que recibieron angiografía coronaria despúes de las 24 h. Grupo 1: Entre 24 - 72 h. Grupo 2: > 72 h.	29 en grupo 1, 47 en grupo 2. El compuesto primario (muerte cardiovascular, infarto miocardio no fatal, falla cardiaca) a 6 meses fue de 13,8% en grupo 1, y 21,3% en grupo 2 (p= 0,661), y a largo plazo (mediana de 57 meses) fue de 37,9% y de 38,3%, respectivamente, (p=0,974), no se encontró diferencia significativa en los resultados individuales.
Coronary angiography after successful thrombolysis - Is the recommended time interval of 24 h an important issue? (2016) ^13^	Estudio retrospectivo. Pacientes fibrinolizados con éxito. Grupo A: Pacientes que no recibieron angiografía coronaria. Grupo B: Pacientes que recibieron angiografía coronaria en las primeras 24 h. Grupo C: Pacientes que recibieron angiografía coronaría después de las primeras 24 h.	278 en el grupo A, 127 en el grupo B, 660 en el grupo C. Grupo A presentó mayor prevalencia de muerte intrahospitalaria (19,8%), frente al grupo B y C (1,6% y 1,4% respectivamente). La regresión logística revelo que a) la no realización de angiografía coronaria después de la fibrinolisis fue un predictor independiente de mortalidad intrahospitalaria b) la ejecución de la angiografía despúes del tiempo recomendado no fue asociado con mayor mortalidad.

En la guía canadiense citada por Britto ^1^, se recomienda fuertemente completar la estrategia farmacoinvasiva antes de las 24 h. Por otro lado, recalcamos que esta no menciona ninguna evidencia sobre su recomendación de que «algunas regiones, que no tengan los recursos requeridos para transferir a todos los pacientes tempranamente después de una fibrinolisis, puedan necesitar transferir solo pacientes de alto riesgo»; además, esta no excluye que los pacientes que no hayan podido ser referidos tempranamente, en las primeras 24 h, no puedan ser derivados posteriormente ^18^.

Con relación a la guía argentina mencionada y su recomendación condicional a favor de un manejo conservador, como una estrategia segura en pacientes sin criterios de «alto riesgo clínico» tras una fibrinolisis exitosa y que no tengan acceso fácil a una sala de hemodinámica, consideramos que se debe ser prudente sobre esta decisión, ya que según el mismo panel la calidad de la evidencia donde se sugiere esta recomendación es baja, subrogada, con alto riesgo de sesgo y no desestiman ni establecen ninguna práctica. Los cinco ensayos del metanálisis de D’Souza (2011) tomados en su subanálisis, involucran un número limitado de pacientes, no incluyeron exclusivamente pacientes de «bajo riesgo», no tuvieron el objetivo de evaluar sus resultados según la estratificación de riesgo del paciente, no todos los estudios hicieron un seguimiento a largo plazo, y en general lo que mostró este metanálisis fueron peores resultados combinados (recurrencia de isquemia y reinfarto) con una estrategia solo conservadora frente a una estrategia invasiva posterior a la fibrinólisis ^19^.

Es conocido que la audiencia objetivo de las guías de la AHA/ACC es más homogénea, y comprende menor diversidad social, económica y política con relación a la guía europea, pero ambas concuerdan que es más beneficioso llevar a cabo la ICP tras la fibrinolisis de forma rutinaria y sin hacer distinción entre pacientes ^3^^,^^20^^,^^21^; asimismo, la guía NICE recomienda considerar la angiografía coronaria durante la misma admisión hospitalaria para aquellos que estan estables tras una fibrinolisis exitosa sin considerar otros criterios ^22^.

Finalmente, tomando en cuenta nuestras altas cifras de morbimortalidad por esta patología a nivel nacional ^23^, superiores a las de otros países de la región, sugerimos que nuestras autoridades y gestores de salud sigan desarrollando estrategias a nivel nacional para mejorar el proceso de atención del infarto de miocardio con elevación del segmento ST, implementando un sistema de redes para la atención del infarto, fortaleciendo la estrategia farmacoinvasiva oportuna en centros periféricos sin capacidad de ICP, y mejorando la capacidad de ICP primaria en los principales hospitales de nuestro país ^24^^,^^25^.

## Financiamiento:

Ninguno
